# Client-Oriented Blind Quality Metric for High Dynamic Range Stereoscopic Omnidirectional Vision Systems

**DOI:** 10.3390/s22218513

**Published:** 2022-11-04

**Authors:** Liuyan Cao, Jihao You, Yang Song, Haiyong Xu, Zhidi Jiang, Gangyi Jiang

**Affiliations:** 1 Faculty of Information Science and Engineering, Ningbo University, Ningbo 315211, China; 2 College of Science and Technology, Ningbo University, Ningbo 315300, China; 3 Zhejiang Provincial United Key Laboratory of Embedded Systems, Hangzhou 310032, China; 4 School of Mathematics and Statistics, Ningbo University, Ningbo 315211, China

**Keywords:** high dynamic range (HDR), HDR stereoscopic omnidirectional vision system, image quality assessment, visual perception

## Abstract

A high dynamic range (HDR) stereoscopic omnidirectional vision system can provide users with more realistic binocular and immersive perception, where the HDR stereoscopic omnidirectional image (HSOI) suffers distortions during its encoding and visualization, making its quality evaluation more challenging. To solve the problem, this paper proposes a client-oriented blind HSOI quality metric based on visual perception. The proposed metric mainly consists of a monocular perception module (MPM) and binocular perception module (BPM), which combine monocular/binocular, omnidirectional and HDR/tone-mapping perception. The MPM extracts features from three aspects: global color distortion, symmetric/asymmetric distortion and scene distortion. In the BPM, the binocular fusion map and binocular difference map are generated by joint image filtering. Then, brightness segmentation is performed on the binocular fusion image, and distinctive features are extracted on the segmented high/low/middle brightness regions. For the binocular difference map, natural scene statistical features are extracted by multi-coefficient derivative maps. Finally, feature screening is used to remove the redundancy between the extracted features. Experimental results on the HSOID database show that the proposed metric is generally better than the representative quality metric, and is more consistent with the subjective perception.

## 1. Introduction

Virtual reality (VR) technologies can provide users with a unique immersive experience with their high resolution, high reproducibility, and full-field viewing [1,2,3]. As one of the most important carriers of VR systems, stereoscopic omnidirectional visual signals can provide users with 360 × 180° field of view (FoV) immersion, binocular perception and view interaction [4,5]. In stereoscopic omnidirectional imaging with the FoV of 360 × 180°, its illumination intensity of the scene is usually very different. Thus, high dynamic range (HDR) stereoscopic omnidirectional vision systems, combining HDR imaging [6,7] and stereoscopic omnidirectional imaging technologies, can better describe real information of a scene. In such a system, a HDR stereoscopic omnidirectional image (HSOI) may suffer distortion during its generation, encoding/transmission and visualization with head-mounted display (HMD), which results in degradation of HSOI quality. Therefore, it is more challenging to establish efficient blind HSOI quality metrics.

Generally, related to the research of stereoscopic omnidirectional image quality assessment (SOIQA), quality estimation has undergone 2D image quality assessment (2D-IQA), stereoscopic image quality assessment (SIQA) and omnidirectional image quality assessment (OIQA). Visual content quality metrics can be divided into full-reference, reduced-reference and blind/no-reference, according to the usage of the reference image information. For 2D-IQA, the classic full reference quality metrics include peak signal-to-noise ratio (PSNR), structural similarity (SSIM) [8], and so on. When a reference image is not available, a blind quality metric is required, and some representative metrics have been proposed, such as OG-IQA [9], GWH-GLBP [10], BRISQUE [11], IL-NIQE [12], dipIQ [13], BMPRI [14], SISBLIM [15], and so on. Moreover, some deep learning based 2D-IQA metrics have also been proposed [16,17]. 

For SIQA, Zhang et al. [18] proposed a full reference SIQA metric with multiscale perceptual features and genetic algorithm training-based support vector machine regression. Jiang et al. [19] proposed an SIQA metric by learning non-negative matrix factorization-based monocular perception and binocular interaction-based color visual features. Liu et al. [20] used a spatial activity model for weighting a cyclopean image of a stereoscopic image pair, and extracted the corresponding features to form an S3D integrated quality (SINQ) metric. Chen et al. [21] considered binocular perception and disparity information, and applied cyclopean images to design an SIQA metric. Li et al. [22] established a two-channel convolutional neural network (CNN) to simulate binocular fusion and binocular competition for SIQA. Meng et al. [23] combined a visual intersection model, multiscale information fusion model and attention-simulated binocular fusion model to design an SIQA metric.

The above 2D-IQA and SIQA metrics are designed for traditional 2D/3D images, which do not take visual perception of omnidirectional images (OIs) and stereoscopic omnidirectional image (SOI) quality assessment into account. For OIQA, initially, some full-reference OIQA metrics based on PSNR and SSIM were proposed. After that, starting from representations of OI, Zheng et al. [24] proposed a segmented spherical projection-based blind OIQA metric (called SSP-OIQA), in which the bipolar and equatorial regions of OI are obtained by the segmented spherical projection, and different feature extraction schemes are designed for evaluating distorted OI. Jiang et al. [3] proposed a perception-driven blind OIQA framework based on cubemap projection (CMP). Considering the HMD viewport viewing mode of OI and the effectiveness of depth learning in visual computing, Sun et al. [25] designed a multi-channel CNN for blind OIQA, which uses six parallel ResNet-34 networks to process viewport images and a quality regression to predict the quality score of distorted OIs. Li et al. [26] proposed an OI-oriented attentive deep stitching method and presented an attention-driven OIQA metric with global and local measures. Fu et al. [27] designed an adaptive hypergraph convolution network for OIQA, which consists of a multi-level viewport descriptor and modeling the viewport interaction through a hypergraph.

For SOIQA, Qi et al. [28] considered perception factors such as the viewport, user behavior and binocular perception, and proposed a viewport perception-based blind SOIQA metric, which mainly includes a binocular perception model and an omnidirectional perception model. Zhou et al. [29] proposed an SOIQA metric based on projection-invariant features and visual saliency; they combined the visual saliency model of chrominance and contrast perception factors to improve the prediction accuracy. Xu et al. established a stereoscopic omnidirectional image database (named SOLID) and proposed a multiple viewports-based full-reference SOIQA metric [30], in which they used the difference map between left and right views to estimate depth perception related features. Chen et al. [4] further proposed a full-reference SOIQA metric based on predictive coding theory. In [31], deep learning was used to design an SOIQA metric.

To visualize HDR images on the displays with standard dynamic range (SDR), an efficient approach is to perform tone-mapping (TM) on HDR images, but this may result in the corresponding degradation in visual quality. Regarding this issue, Gu et al. [32] designed a blind tone-mapped quality index (BTMQI), which combined information entropy, structure and natural scene statistics for quality evaluation. Jiang et al. [33] analyzed texture distortion and color distortion of different brightness regions in tone-mapped HDR images, and designed a blind tone-mapped image quality assessment (BTMIQA) metric that considered the details of the bright and dark regions, as well as their naturalness and aesthetics features for quality evaluation. Fang et al. [34] proposed a tone-mapped HDR image quality metric with gradient and color difference statistics, which used the sensitivity of human eyes to image structure changes to measure image degradation, and used local binary pattern to describe color distortion. Yue et al. [35] presented a tone-mapped HDR image quality metric by extracting three quality-sensitive features, namely color, naturalness and structure. Zhao et al. [36] extracted features of a tone-mapped HDR image from pixel domain, sharpness and chromaticity for predicting the quality of the tone-mapped HDR image.

HSOI quality assessment (HSOIQA) involves not only binocular perception and OI perception, but also HDR/TM perception. Up until now, HSOIQA has been an unstudied and challenging issue. To solve this issue, in this paper, a client-oriented blind HSOIQA metric based on visual perception is proposed, which includes two main modules, that is, a monocular perception module (MPM) and a binocular perception module (BPM). In the MPM, the global color distortion, symmetric/asymmetric distortion and scene distortion are characterized. In the BPM, new feature extraction schemes of binocular fusion map and a binocular difference map based on joint image filtering are designed. The corresponding features are extracted by brightness segmentation of the binocular fusion map, and the natural statistical features are extracted from the binocular difference map. All viewport image-based features are aggregated according to the significance, and feature screening is performed and an objective quality score of HSOI is predicted. Experimental results show that the proposed metric outperforms the representative blind quality metrics. The main contributions of this paper are as follows:

(1) A client-oriented blind HSOIQA metric based on visual perception is established for the client’s distorted HSOI in HSOV system, which combines binocular perception, OI perception and HDR/TM perception.
(2) New feature extraction schemes of the binocular fusion map and the binocular difference map based on joint image filtering are designed for efficiently evaluating the quality of distorted HSOI.
(3) In feature extraction, the information expression and perception capabilities of HSOIs at different resolutions are further explored with multiscale computing methods.

The rest of this paper is arranged as follows. Section 2 describes the proposed metric for HSOIs at the client of the HSOV system. Section 3 gives experimental results and analyses. Finally, Section 4 concludes the paper.

## 2. The Proposed Metric

This section states the research ideas from the perspective of visual perception and gives an overview of the proposed metric; then, the proposed metric is described in detail.

### 2.1. Overview of the Proposed Metric

Generally, the HSOV system consists of processes such as HSOI generation, encoding/decoding with JPEG XT/H.265 and visualization by using a head mounted display (HMD) with SDR. The processes may produce corresponding distortion, such as encoding distortion, TM distortion and mixed distortion, resulting in degradation of the quality of the user’s visual experience.

The human binocular vision system has two visual pathways: the dorsal pathway and the ventral pathway [37,38,39]. The dorsal pathway starts from the primary visual cortex V1 area, and flows through the V2, V3 and V5 areas; its function is to complete the guidance from visual information to action [38]. The ventral pathway starts from the V1 area, and flows through V2, V3 and V4 areas to complete the perception and recognition of visual behaviors [39]; it is also related to long-term memory. For visual content quality assessment, visual perception of distortion is extremely important, so the ventral pathway with the V1, V2, V3 and V4 areas has a certain guiding significance for perceptual quality assessment. In the visual cortex, there are two types of cells: simple cells and complex cells [40]. The simple cells process the retinal information of the left and right views received from the corresponding lateral geniculate nucleus. The complex cells connect the left and right view signals with the binocular signals. Specifically, the V1 area corresponds to the simple features such as luminance, chromaticity, edge, spatial frequency, and the V2 area will recognize higher level features, such as texture and shape, in addition to transmitting lower level features of the V1 area. The V3 and V4 areas belong to the occipital cortex and actually have little correlation with perceptual quality, but they can further encode complex image features. The perception process of the V1, V2, V3 and V4 areas provides a theoretical basis for the subsequent modeling of HSOIQA.

**Figure 1:**
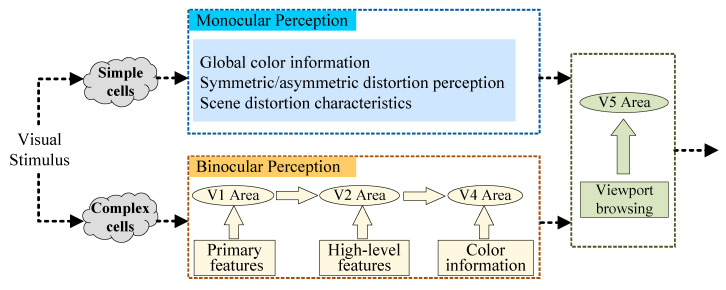
The visual perception process of user viewing HSOIs.

Here, based on the human visual system (HVS), the distorted HSOI is input as a visual stimulus of HVS to simulate the left view/right view information processed by simple cells in the V1 area to model the monocular perception of HSOI. At the same time, it also simulates the complex cell processing process to model the binocular perception effect, as shown in Figure 1. For the monocular perception modeling of the distorted HSOI, it combines the symmetric/asymmetric encoding distortion characteristics, OI viewport characteristics and scene characteristics (outdoor/indoor/night scenes) of distorted HSOI to extract global color information, symmetric/asymmetric distortion perception and scene distortion perception, respectively. For the binocular perception modeling of distorted HSOI, the information transfer mechanism of the V1, V2, V3 and V4 areas is simulated. Combined with the ventral pathway, primary features such as brightness are first perceived in the V1 area; when the information from the V1 area is transferred to the V2 area, higher-level features are recognized. Thereafter, color information is perceived in the V4 area. In addition, when the user wears HMD to browse the image content in the current viewport, the image content may guide the user’s behavior in selecting the next viewport to be browsed. For example, when the image browsed in the current viewport is incomplete, the user is very likely to rotate their head to observe next viewport to browse the complete image content. This process of actively selecting the viewport for browsing will be completed in the V5 area. After the next viewport is selected, the above process will be repeated until the user completes their viewing of the entire HSOI.

**Figure 2:**
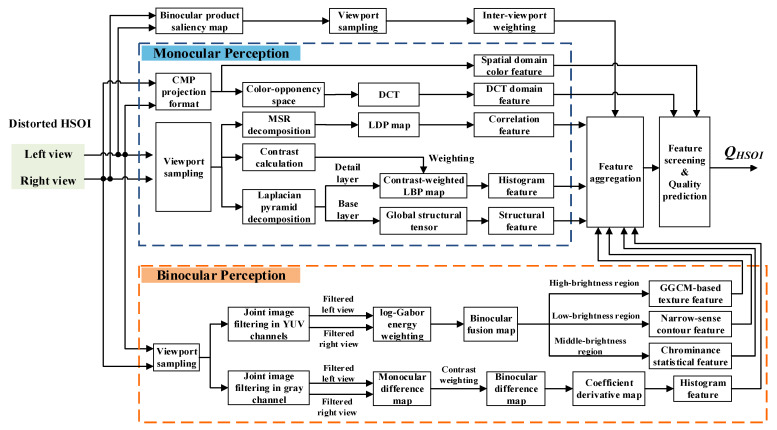
The framework of the proposed HSOIQA metric for the client in the HSOV system.

Based on the above analysis, for client-oriented HSOIQA in the HSOV system, we propose a visual perception-based blind HSOIQA metric, as shown in Figure 2. The proposed metric mainly includes viewport sampling, a monocular perception module (MPM), a binocular perception module (BPM), feature screening and quality regression. For the MPM, firstly, HSOI is transformed from an equirectangular projection (ERP) format to CMP format, and then the global color features are extracted in the spatial and discrete cosine transform (DCT) domains. Secondly, considering the unique symmetric/asymmetric distortion of HSOI, its distorted left and right views are measured by multiscale retinex (MSR) decomposition. Then, combining with a Laplacian pyramid decomposition model, the distortions of the different scenes with indoor/outdoor and day/night are measured from the characteristics of contrast, detail and structure. For the BPM, based on joint image filtering, a binocular fusion map is generated to represent the similarity of the HSOI’s left and right views; the brightness-based viewport image is further segmented to distinguish perceptual characteristics of different brightness regions, which is consistent with the information transmission process of the V1, V2 and V4 areas in the human visual system. The calculated binocular difference map represents the difference information between left and right views. Before quality regression, all the extracted feature vectors are processed by feature screening. Finally, quality regression with a random forest model is used to predict the objective quality score of distorted HSOI.

### 2.2. Viewport Sampling

Let ***I****_HSOI_* be the HSOI signal output from the HSOV system to user’s HMD, which may suffer encoding distortion, TM distortion and mixed distortion, where ***I****_HSOI_* = {***I****_L_*, ***I****_R_*}, ***I****_L_* and ***I****_R_* represent the left and right views of HSOI, respectively. ***I****_HSOI_* can be represented by the ERP format, CMP format, spherical format and viewport images, respectively.

At the client of HSOV system, the user can actively select a viewport according to the content of the HSOI through HMD with SDR. For HSOIQA, the HSOI can be divided into the equatorial region and bipolar regions from the perspective of the user’s behavior. Let *M* be the number of viewports uniformly sampled in the equatorial region, and the bipolar regions are sampled according to the significance of the binocular product, and one viewport is taken for each polar region; thus, the total number of viewports is *M* + 2.

Assuming that the vertical FoV of a viewport is *φ*, the equatorial region corresponds to the latitude range of [−*φ*/2, *φ*/2], and the bipolar regions correspond to the latitude ranges of (*φ*/2, 90°] and [−90°, −*φ*/2), respectively. If the vertical FoV angle of HMD device is 110°, *φ* will be set to 110°. In the equatorial region, *M* viewports are uniformly sampled at equiangular intervals with the angle set to 2π/*M*. For the bipolar regions, the position with the largest pixel value in the corresponding binocular product saliency map ***S****^LR^* is selected as the center of the viewport. ***S****^LR^* is obtained as follows: (1) For the left and right views ***I****_L_* and ***I****_R_*, their saliency maps ***S****^L^* and ***S****^R^* are computed by the method in [41], respectively; (2) ***S****^LR^* is viewed is the correlation measure between ***S****^L^* and ***S****^R^* after stereoscopic matching, ***S****^LR^* = {*S^LR^*(*i*,*j*)}, and expressed as follows:

*S^LR^*(*i*,*j*) = *S^L^*(*i*,*j*) × *S^R^*((*i*,*j*) + *d_i,j_*) (1)

where *d_i,j_* denotes the disparity of ***S****^R^* relative to ***S****^L^* on the pixel at the position of (*i*,*j*), which is calculated by the optical flow method in [42].

### 2.3. Monocular Perception Module (MPM) for Distorted HSOI

This subsection extracts the monocular perceptual features of the distorted HSOI from three aspects: global color information, symmetric/asymmetric encoding distortion perception, and scene distortion perception. Among them, the color information is a subjective overall perception, and all viewport images need to be reconstructed first when viewed by users, so, the CMP format of ***I****_HSOI_* is used for global color feature extraction. For symmetric/asymmetric distortion perception and scene distortion perception, the perceptual features are extracted based on the viewport images.

(1) Global color feature extraction

As the output at the client of HSOV system, the distorted HSOI may consist of encoding distortion, TM distortion and the mixed distortion. Compared with the ERP representation of ***I****_HSOI_*, its CMP representation more easily describes the monocular distortion of HSOI. Furthermore, numerous studies have shown that color information is processed with color-opponency in the human visual system. Therefore, the global color features are extracted in the spatial domain and DCT domain, respectively, based on the color-opponency space.

According to the work of Hasler et al. [43], the color in an image is quantized to qualitatively process or code the impact on color visually. Here, taking the left view ***I****_L_* of ***I****_HSOI_* as an example, ***I****_L_* = (***R****_L_*, ***G****_L_*, ***B****_L_*). Its CMP format can be expressed as ***I****_L_* = {***I****_Li_*, *i =* 1, 2,…, 6}, ***I****_Li_* is the *i*-th face of the six faces in the CMP format. First, ***I****_L_* is converted from RGB space to the red–green and yellow–blue opponency channels, denoted as ***ρ****_L_*_rg_ and ***ρ****_L_*_yb_, ***ρ****_L_*_rg_ = ***R****_L_ − **G**_L_*, ***ρ****_L_*_yb_ = 0.5(***R****_L_* + ***G****_L_*) − ***B****_L_*. Let *μ_L_*_rg_ and *μ_L_*_yb_ denote the mean of ***ρ****_L_*_rg_ and ***ρ****_L_*_yb_, and *σ_L_*_rg_ and *σ_L_*_yb_ denote the variance of ***ρ****_L_*_rg_ and ***ρ****_L_*_yb_; then, two statistic features *μ*^2^*_L_*_rg−*L*yb_ and *σ*^2^*_L_*_rg−*L*yb_ of ***ρ****_L_*_rg_ and ***ρ****_L_*_yb_ are expressed as follows:

*μ*^2^*_L_*_rg−*L*yb_ = *μ*^2^*_L_*_rg_ + *μ*^2^*_L_*_yb_ (2)

*σ*^2^*_L_*_rg−*L*yb_ = *σ*^2^*_L_*_rg_ + *σ*^2^*_L_*_yb_ (3)

The most intuitive TM operators (TMOs) are generally to change the mean value of the pixel value distribution, and then change the degree of numerical dispersion of pixels. Therefore, the joint statistical measure *J_L_*_rg−*L*yb_ of ***ρ****_L_*_rg_ and ***ρ****_L_*_yb_ is expressed to describe the spatial color feature of ***I****_L_*, and calculated as follows [43]:

*J_L_*_rg−*L*yb_ = *σ_L_*_rg−*L*yb_ + 0.3*μ_L_*_rg−*L*yb_ (4)

Similarly, for the right view ***I****_R_* of ***I****_HSOI_*, its joint statistical measure *J_R_*_rg−*R*yb_ can also be obtained. Thus, the global spatial color feature ***F****_CS_* is defined for the distorted HSOI, ***F****_CS_* = (*J_L_*_rg−*L*yb_, *J_R_*_rg−*R*yb_).

**Figure 3:**
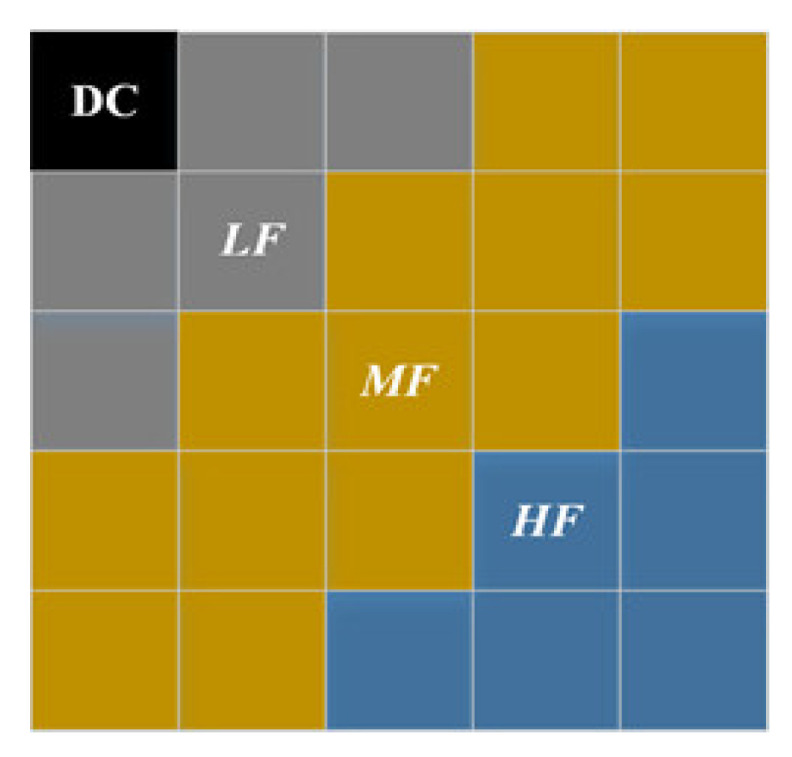
Classification of *N_u_ × N_v_* DCT coefficients.

Then, the color features in the transform domain are extracted. Taking the CMP format of ***I****_L_* as an example, for its two color-opponency channels, ***ρ****_L_*_rg_ and ***ρ****_L_*_yb_, their non-overlapping *N_u_ × N_v_* blocks are transformed with DCT. Let {*ξ_L_*_,*k*_(*u*,*v*); *u* = 1, 2,…, *N_u_*, *v* = 1, 2,…, *N_v_*} denote DCT coefficients of a block, where *k* represents the color antagonist channel ***ρ****_L_*_rg_ or ***ρ****_L_*_yb_; here, *N_u_* and *N_v_* are set to 5. For {*ξ_L_*_,*k*_(*u*,*v*)}, its DC component is discarded, and its AC components are divided into three frequency bands: low frequency (LF), middle frequency (MF) and high frequency (HF) as shown in Figure 3. The variance of the three frequency bands of each image block is calculated separately as the band energy feature, the mean of the three frequency bands’ variances of all image blocks is considered as the final energy feature of the corresponding frequency band; then, the energy features of six faces of the CMP format of ***I****_L_* are averaged. For two color-opponency channels, ***ρ****_L_*_rg_ and ***ρ****_L_*_yb_, of ***I****_L_*, 6-dimensional features can be extracted. Similarly, for ***ρ****_R_*_rg_ and ***ρ****_R_*_yb_, of ***I****_R_*, 6-dimensional energy features can also be obtained, which constitutes 12-dimensional color features in the DCT domain, ***F****_CD_*, of HSOI.

Finally, the global color features of HSOI are denoted as ***F****_CE_*, ***F****_CE_* = (***F****_CS_*, ***F****_CD_*).

(2) Symmetric/asymmetric encoding distortion measure

Different from 2D image coding, a stereoscopic image can be encoded with asymmetric encoding by using different quantization parameters (QPs) for its left and right views, so as to improve the encoding efficiency by taking the binocular masking effect of human eyes. The distortion-level difference between the left and right views has a great impact on the quality of user’s experience to the encoded stereoscopic image. Here, a correlation measure between the left and right views is designed to evaluate the information difference caused by different distortion levels of the left and right views.

For ***I****_L_* and ***I****_R_*, viewport sampling is first performed to obtain the corresponding left and right viewport image sets {***V****_L_*_,*m*_} and {***V****_R_*_,*m*_}, respectively, where *m* = 1, 2,…, *M* + 2. From the multi-resolution perception of the human visual system, in the process of gradually reducing the image resolution from high to low, the focus of the human eyes shifts from fine textures to rough structures. MSR decomposition [44] is used for image preprocessing in this work. The complementary information of different scales can effectively detect the image content that is not easy to find at a single scale.

For a given image, the illumination component can be estimated by MSR decomposition. Taking $V_{L,m}$ as an example, $V_{L,m}=\left\{V_{L,m}\left(x,y\right)\right\}$, its illumination component $\Psi_{L,m}$, $\Psi_{L,m}$ = {$\Psi_{L,m}\left(x,y\right)\}$ can be calculated as follows:

(5) $$\Psi_{L,m}\left(x,y\right)=V_{L,m}\left(x,y\right)\;⊗\;g(x,y)$$

where ⨂ is convolution operation, *g*(*x*,*y*) is Gaussian function, *g*(*x*,*y*) = *N_g_exp*(−(*x*^2^ + *y*^2^)/*η*), *N_g_* is normalization factor; *η* is the scale parameter of Gaussian function. When the value of *η* is large, the detail recovery is coarse, and when the value is small, the detail recovery is fine. Here, in order to reflect the multiscale characteristics, three scale factors with significant differences were used: small, medium and large, *η* can be set to one element of {*η*_1_, *η*_2_, *η*_3_}.

MSR decomposition can be used to describe illumination features by three different scale filtering on the image and then weighted summation; here, the gray-scale images of the viewport’s left and right views are directly processed to obtain the corresponding illumination components with different *η* (*η* ∈ {*η*_1_, *η*_2_, *η*_3_}), which are, respectively, denoted as $\Psi_{L,m}$ and $\Psi_{R,m}$, where $\Psi_{L,m}$ = {$\Psi_{L,m}^{\;\eta }$} and $\Psi_{R,m}$ = {$\Psi_{R,m}^{\;\eta }$}.

**Figure 4:**
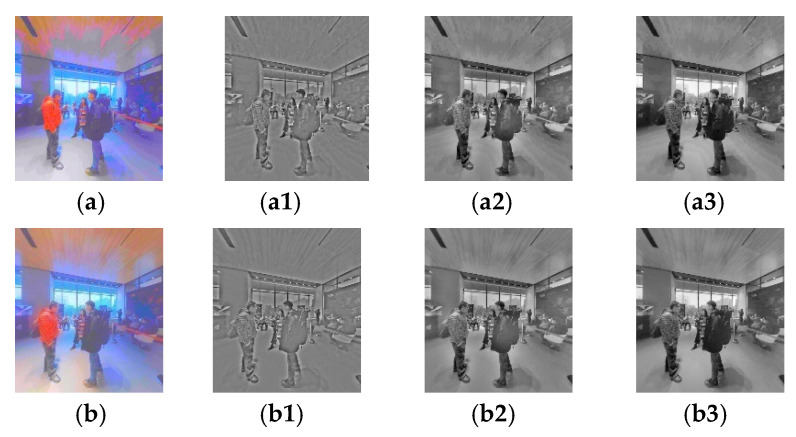
An example of MSR decomposition of the distorted HSOI in the HSOID database [45] at the client of the HSOV system (*η* ∈ {*η*_1_, *η*_2_, *η*_3_}). (**a**) Left view of the distorted viewport; (**a1**–**a3**) are three scale illumination components of (**a**), $\Psi_{L,m}^{\eta }$; (**b**) Right view of the distorted viewport; (**b1**–**b3**) are three scale illumination components of (**b**), $\Psi_{R,m}^{\eta }$; (from left to right, *η*_1_ = 25, *η*_2_ = 100, *η*_3_ = 240).

Figure 4 shows an example of MSR decomposition of distorted HSOI in the HDR stereoscopic omnidirectional image database (HSOID) [45] at the client of the HSOV system (here, *η*_1_ = 25, *η*_2_ = 100, *η*_3_ = 240). The original HSOI at the server in the HSOV system is encoded with an asymmetric encoding distortion level of (L1, L3), i.e., the encoding distortion level of the left view is L1, and the encoding distortion level of the right view is L3. To visualize the compressed HSOI on HMD with SDR, DurandTMO [46] is used in TM processing. It can be found as follows:

(i) For $\Psi_{L,m}$ and $\Psi_{R,m}$, their MSR decomposition with *η*_1_ can show more details than those of MSR decomposition with *η*_2_ and *η*_3_, especially in the window regions in Figure 4. It indicates that the image’s MSR decomposition with different *η* values contains different information, and three-scale MSR decomposition can complement each other.
(ii) Compared with the viewport’s left view, the distortion level of the viewport’s right view is lower, and its block effect is less, $\Psi_{R,m}$ has a clearer texture than that of $\Psi_{L,m}$, especially in the ceiling and ground regions. It indicates that MSR decomposition can reflect different distortion characteristics of the left and right views with different distortion levels to a certain extent.

For $\Psi_{L,m}$ and $\Psi_{R,m}$, their feature maps are further calculated. First, de-mean normalization is performed on them. Second, a local derivative pattern (LDP) [47] is used to measure the texture information of $\Psi_{L,m}$ and $\Psi_{R,m}$ after obtaining the second derivative of $\Psi_{L,m}$ and $\Psi_{R,m}$ in four directions of {0°, 45°, 90°, 135°}. The LDP map in each direction can be quantized by a 10-dimensional histogram according to the rotation-invariant uniform local binary pattern. After the above operations, the quantized LDP histograms of $\Psi_{L,m}$ and $\Psi_{R,m}$ are obtained, and respectively expressed as $H_{L,m}^{\eta }=\{h_{L,m}^{\eta ,1-10},h_{L,m}^{\eta ,11-20},h_{L,m}^{\eta ,21-30},h_{L,m}^{\eta ,31-40}\}$ and $H_{R,m}^{\eta }=\{h_{R,m}^{\eta ,1-10},h_{R,m}^{\eta ,11-20},h_{R,m}^{\eta ,21-30},h_{R,m}^{\eta ,31-40}\}$, *η* ∈ {25, 100, 240}. The correlation coefficient can be used to measure the correlation degree between two random variables, and its value range is [−1, 1]; the larger the absolute value of the correlation coefficient, the higher the correlation between the two. Then, based on $H_{L,m}^{\eta }$ and $H_{R,m}^{\eta }$, the absolute correlation coefficient *C_A_* and correlation distance *C_D_* of each 10-dimensional histogram are calculated as the similarity features of the left and right views, *C_A_* and *C_D_* are computed as $C_{A}=\left|\mathrm{corr}(H_{L,m}^{\eta },H_{R,m}^{\eta })\right|$ and $C_{D}=\left|pdist(H_{L,m}^{\eta },H_{R,m}^{\eta })\right|$, where *corr*(·) represents the correlation coefficient function, *pdist*(·) represents the correlation distance function, and |·| represents the absolute operation.

Finally, the absolute correlation coefficients and correlation distances of Gaussian functions with three scale parameters are taken as the symmetric/asymmetric encoding distortion feature vector ***f_cco__r_***.

Taking the scenarios in the HSOID [45] as examples, Table 1 shows *C_A_* and *C_D_* of the distorted viewport’s left and right views processed by DurandTMO under encoded with 9 distortion levels (the first 5 are asymmetric encoding distortion, and the last 4 are symmetric encoding distortion). In addition, Table 2 shows the relationship between *C_A_* and correlation degree, which reflects the correlation degree of the left and right views with different distortion levels. It can be found as follows:

(i) The correlation degrees of the distorted viewport’s left and right views, encoded with 5 asymmetric distortion levels, are obviously different. Although the asymmetric distortion levels, (L1, L3), (L3, L4) and (L2, L3), can be judged as very strong correlation degrees, their absolute correlation coefficients *C_A_* are distributed in different levels; the first two are in the range of 0.8 to 0.9, while the latter are in the range of 0.9 to 1.0. It shows that *C_A_* and *C_D_* can effectively distinguish different levels of asymmetric distortion.
(ii) The correlation degrees of the distorted viewport’s left and right views, encoded with 4 symmetrical distortion levels, are all very strongly correlated, and generally their *C_A_* values tend to be larger as the distortion level is lower.
(iii) The *C_A_* values of the distorted viewports, encoded with the symmetrical distortion levels, are generally larger than those with the asymmetrical distortion levels; it indicates that *C_A_* and *C_D_* can effectively distinguish the types of symmetrical/asymmetrical encoding distortion and their degree of distortion to a certain extent.

(3) Feature extraction with scene analysis

The HSOID [45] includes the different scenes of indoor, outdoor, day and night. In the imaging of indoor scenes, most of the light usually comes from the ceiling light, which may not be sufficient for outdoor scenes; at the same time, most of them contain window regions, and the brightness of the window regions is relatively high, which is prone to loss of details in the imaging. In the imaging of outdoor scenes during the daytime, the light is relatively sufficient and the contrast is relatively high. There may be a large region of sky in the outdoor imaging, because the sky region is relatively flat, the block effect caused by encoding distortion is more perceptible. Especially when wearing an HMD to view the HSOI, it can be seen from the near-eye perception that the distortion in this region is more likely to affect the subjective quality. The night scene is generally dark, with relatively low contrast and fuzzy structure.

In summary, based on contrast, detail and structure of an image, feature extraction can be performed to synthesize perceptual distortion features of various scenes. Among them, the details and structures can be represented by the image’s detail layer and base layer in combination with the idea of image decomposition.

To generate the base and detail layers of the image, Laplacian pyramid decomposition [48] can be used. Taking a distorted viewport’s left view ***V****_L_*_,*m*_ as an example, it is decomposed by Laplacian pyramid at three scales, and a total of three detail layers and three base layers are obtained.

For ***V****_L_*_,*m*_ with the resolution of *N_H_* × *N_V_*, its 3-layer detail layer set is expressed as ***D****_L_*_,*m*_ = {***D***^1^*_L_*_,*m*_, ***D***^2^*_L_*_,*m*_, ***D***^3^*_L_*_,*m*_}, and the corresponding resolutions are *N_H_* × *N_V_*, (0.5*N_H_*) × (0.5*N_V_*) and (0.25*N_H_*) × (0.25*N_V_*), respectively. The 3-layer base layer set of ***V****_L_*_,*m*_ is expressed as ***B****_L_*_,*m*_ = {***B***^1^*_L_*_,*m*_, ***B***^2^*_L_*_,*m*_, ***B***^3^*_L_*_,*m*_}, and their resolutions are (0.5*N_H_*) × (0.5*N_V_*), (0.25*N_H_*) × (0.25*N_V_*) and (0.125*N_H_*) × (0.125*N_V_*), respectively. Considering the resolution of the viewport image, three layers of detail layers and the first two layers of the base layers are used as the layers after Laplacian pyramid decomposition, that is, the set of the base layers is denoted as ***B****′_L_*_,*m*_ = {***B***^1^*_L_*_,*m*_, ***B***^2^*_L_*_,*m*_}. The base layers of ***V****_L_*_,*m*_ retain most of the information of ***V****_L_*_,*m*_, while the detail layers of ***V****_L_*_,*m*_ show the detail information; and the higher the resolution, the finer the level of detail displayed.

After the above preprocessing of the distorted viewport’s left and right views, the detail layer sets, ***D****_L_*_,*m*_ and ***D****_R_*_,*m*_, and the base layer sets, ***B****′_L_*_,*m*_ and ***B****′_R_*_,*m*_, can be obtained. First, the detail features are extracted based on ***D****_L_*_,*m*_ and ***D****_R_*_,*m*_. Considering that a local binary pattern (LBP) [49] can describe spatial domain information by encoding the spatial position relationship between the center pixel and its neighbor pixels within a certain radius, different patterns can characterize structures such as points, lines, and edges, and a contrast-weighted LBP (CLBP) is adopted in combination with the contrast information. Taking ***D****_L_*_,*m*_ as an example, its LBP can be expressed as follows:

(6) $$LBP_{P,R}=\sum_{i=0}^{P-1}T_{h}(D_{L,m,i}-D_{L,m,c})\cdot 2^{i}$$

where *P* is the number of neighborhoods, and *R* is the neighborhood radius, *P* and *R* are set to 8 and 1 in [49]. *D_L_*_,*m,c*_ and *D_L_*_,*m,i*_ represent the values of the pixels in ***D****_L_*_,*m*_ and the pixels in the neighborhood centered on ***D****_L_*_,*m*_, respectively. *T_h_*(·) is the threshold function, and expressed as follows:

(7) $$T_{h}(D_{L,m,i}-D_{L,m,c})=\left\{\begin{array}{cc} 1, & \mathrm{if}\;(D_{L,m,i}-D_{L,m,c})\geq 0\\ 0, & \mathrm{otherwise} \end{array}\right.$$

The rotation invariant uniform LBP is expressed as follows:

(8) $$LBP_{P,R}^{riu2}=\left\{\begin{array}{cc} \sum_{i=0}^{P-1}T_{h}(D_{L,m,i}-D_{L,m,c}), & \mathrm{if}\;u({\mathrm{LBP}}_{P,R})\leq 2\\ P+1, & otherwise \end{array}\right.$$

where the superscript ‘riu2’ reflects the use of rotation invariant uniform patterns that have *u* value less than 2, and *u*(·) is the uniform metric, and expressed as follows:

(9) $$u({\mathrm{LBP}}_{P,R})=\left\|T_{h}(D_{L,m,P-1}-D_{L,m,c})-T_{h}(D_{L,m,0}-D_{L,m,c})\right\|+\sum_{i=0}^{P-1}\left\|T_{h}(D_{L,m,i}-D_{L,m,c})-T_{h}(D_{L,m,i-1}-D_{L,m,c})\right\|$$

Generally, the rotation invariant uniform LBP has *P* + 2 modes, and each mode represents different image content information. Let *k* be the mode index. Histogram of CLBP is expressed as follows:

(10) $$h_{CLBP}(k)=\sum_{i=1}^{N}C_{i}f(LBP_{P,R}(i),k)$$

(11) $$f(x,y)=\left\{\begin{array}{cc} 1, & \mathrm{if}\;x=y\\ 0, & \mathrm{otherwise} \end{array}\right.$$

where *N* is the number of pixels of an image in ***D****_L_*_,*m*_, ***C*** is the contrast map of ***V****_L_*_,*m*_, ***C*** = ***σ****_c_*/(***μ****_c_* + *ε*), *C_i_* is the *i*-th pixel’s value in ***C***, ***C*** = {*C_i_*}; ***μ****_c_* and ***σ****_c_* are the mean map and standard deviation map of ***V****_L_*_,*m*_, and *ε* is a constant that prevents the denominator from being 0, and is set to 0.00001.

Because *P* and *R* are set to 8 and 1, respectively, 30-dimensional histogram features of ***D****_L_*_,*m*_ are obtained after the above operations. Similarly, 30-dimensional histogram features of ***D****_R_*_,*m*_ can also be obtained. Finally, the detailed features of the distorted left and right views are expressed as ***f****_CLBP_*.

Then, structural features are extracted from the base layer sets ***B****′_L_*_,*m*_ and ***B****′_R_*_,*m*_. Here, the global structural tensor is used for extracting the base layer’s structural features. Taking ***B***^1^*_L_*_,*m*_ in ***B****′_L_*_,*m*_ = {***B***^1^*_L_*_,*m*_, ***B***^2^*_L_*_,*m*_} as an example, a 2D structural tensor transformation is performed, the structural tensor matrix is decomposed and its two eigenvalues, *λ_L_*_1_ and *λ_L_*_2_, are used to describe the structural features of ***B***^1^*_L_*_,*m*_. For ***B***^2^*_L_*_,*m*_, the eigenvalues of its structural tensor matrix can be also obtained as *λ_L_*_3_ and *λ_L_*_4_.

Similarly, for ***B****′_R_*_,*m*_, the corresponding eigenvalues of the structural tensor matrix can be obtained as *λ_R_*_1_, *λ_R_*_2_, *λ_R_*_3_ and *λ_R_*_4_. Then, the structural features of the distorted left and right views are denoted as ***f****_st_*.

Finally, the feature set extracted by the MPM is the global color feature ***F****_CE_*, symmetric/asymmetric distortion feature ***f****_corr_*, detailed feature ***f****_CLBP_* and structural feature ***f****_st_*.

(4) Feature aggregation with viewport significance

When viewing omnidirectional visual contents, users are usually guided to select viewports by the saliency as regions of interest. The contribution of different viewports to the overall quality needs to be weighted according to their saliency. For binocular product salience map ***S****^LR^*, viewport sampling is performed first to obtain a series of saliency viewport maps, $S_{V}^{LR}=\{S_{V_{1}}^{LR},S_{V_{2}}^{LR},\ldots ,S_{V_{M+2}}^{LR}\}$. The viewport-normalized saliency value ***W****_S_* = {***W****_m_*; *m* = 1, 2,…, *M* + 2} is calculated as the significance weight to express user’s preference for different viewport images, which can be calculated as follows:

(12) $$W_{m}=\sum_{p}S_{V_{m}}^{LR}(p)/\sum_{m=1}^{M+2}\sum_{p}S_{V_{m}}^{LR}(p)$$

where $S_{V_{m}}^{LR}(p)$ is the pixel’s value at the position of *p*.

For the above extracted features ***f****_corr_*, ***f****_CLBP_* and ***f****_st_*, Equation (12) is used to perform feature aggregation on them to obtain the aggregated features, which are, finally, expressed as ***F****_corr_*, ***F****_CLBP_* and ***F****_st_*.

### 2.4. Binocular Perception Module (BPM) for Distorted HSOI

Generally, the human binocular perception has three aspects, the initial stage is binocular fusion; information that cannot be fused leads to binocular competition; in the process of competition, if one view is completely dominant, binocular suppression occurs. The process of binocular perception is a complex physiological mechanism. Both the user’s eyes and brain play a role in this process, and it is difficult for traditional signal processing-based methods to achieve this process through mathematical formulas and derivation. Therefore, current research generally expresses the processes of fusion and competition by simulating biological mechanisms to establish binocular effects for perceptual quality evaluation. Based on joint image filtering, this subsection designs new binocular mechanism modeling schemes, and combines the perceptual characteristics of the V1, V2 and V4 areas of the cerebral cortex for feature extraction.

(1) Joint image filtering

Previous studies [50] showed that the content difference between the left and right views of a stereoscopic image was due to the existence of parallax, but the final result of human binocular perception undergoing three fluctuations is to form a stable stereoscopic image. Therefore, it can be inferred that there is an interactive filtering effect between the left and right views.

(2) Binocular fusion map and feature extraction

The traditional binocular fusion images are mainly realized based on gray-scale images. However, Den Oudenet et al. [51] proved the contribution of color information to binocular matching; it indicates that color information helps solve the binocular matching problem of complex images, and color and brightness information have independent contributions. Thus, for one of the distorted viewport images, (***V****_L_*_,*m*_, ***V****_R_*_,*m*_), it is first converted from RGB space to YUV space. On the channels of Y, U and V, joint image filtering *f_g_*(·) is performed on them, respectively, and the filtered left view of the distorted viewport image is recorded as ***Φ****_L_*_,*m*_ = *f_g_*(***V****_L_*_,*m*_, ***V****_R_*_,*m*_) and ***Φ****_L_*_,*m*_ = {***Φ****^Y^_L_*_,*m*_, ***Φ****^U^_L_*_,*m*_, ***Φ****^V^_L_*_,*m*_}. Similarly, the filtered right view of the distorted viewport image is denoted as ***Φ****_R_*_,*m*_ = *f_g_*(***V****_R_*_,*m*_, ***V****_L_*_,*m*_) and ***Φ****_R_*_,*m*_ = {***Φ****^Y^_Rm_*, ***Φ****^U^_R_*_,*m*_, ***Φ****^V^_R_*_,*m*_}.

Considering that a log-Gabor filter can simulate the multiscale and multi-directional selection characteristics of the receptive field of the visual cortex. Therefore, the log-Gabor filter amplitude responses of ***Φ****_L_*_,*m*_ and ***Φ****_R_*_,*m*_ are calculated as energy factors to further fuse their information. The log-Gabor filter with the scale *s* and direction *o* is expressed as $G_{s,o}(\omega ,\theta )=\exp\left[-\frac{{(\log(\omega /\omega_{s}))}^{2}}{2\delta_{s}^{2}}\right]\cdot \exp\left[-\frac{{\left(\theta /\theta_{o}\right)}^{2}}{2\delta_{o}^{2}}\right]$, where *θ* is the orientation angle, and *δ_s_* and *δ_o_* are the filter strengths; and *ω* and *ω_s_* are the normalized filter radial frequency and the corresponding filter center frequency, respectively.

Let $\left[{\tilde{\psi }}_{s,o},{\tilde{\zeta }}_{s,o}\right]$ be a set of responses of the log-Gabor filter in different directions and scales, then its output amplitude *A* is expressed as the sum of the responses of all scales and directions, and expressed as follows:

(13) $$A=\sqrt{{\left(\sum_{s}\sum_{o}{\tilde{\psi }}_{s,o}\right)}^{2}+(\sum_{s}\sum_{o}{\tilde{\zeta }}_{s,o}{)}^{2}}$$

Calculate the log-Gabor filtering output amplitudes of ***Φ****_L_*_,*m*_ and ***Φ****_R_*_,*m*_, respectively, and denote them as *A_L_*_,*m*_ and *A_R_*_,*m*_. Then, their energy weighting factors, *E_L,m_* and *E_R,m_*, are expressed as follows:

*E_L,m_ = A_L,m_/(A_L,m_ + A_R,m_ + ε)* (14)

*E_R,m_ = A_R,m_/(A_L,m_ + A_R,m_ + ε)* (15)

***Φ****_L_*_,*m*_ and ***Φ****_R_*_,*m*_ are further weighted by *E_L,m_* and *E_R,m_*, respectively. Thus, the fusion image ***Φ****_m_* is expressed as follows:

***Φ****_m_ = E_L,m_ · **Φ**_L,m_ + E_R,m_ · **Φ**_R,m_* (16)

For ***Φ****_m_* = {***Φ****^Y^_m_*, ***Φ****^U^_m_*, ***Φ****^V^_m_*}, the final binocular fusion map ***Φ****′_m_* is generated by using the Y, U and V channels of ***Φ****_m_*.

**Figure 5:**
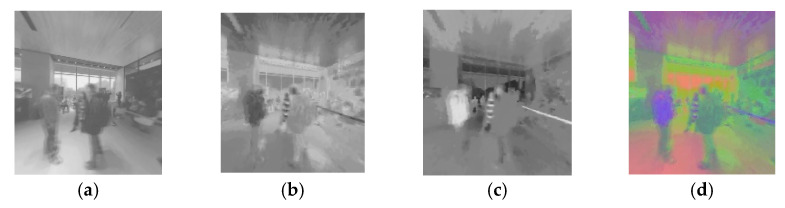
An example of binocular fusion map. (**a**) Y channel, (**b**) U channel, (**c**) V channel, (**d**) three-channel combination with the visualization of pseudo color.

Figure 5 shows an example of binocular fusion map obtained by taking Figure 4 as the test viewport images, including the single-channel fusion map of the Y, U, or V channel and the fusion map ***Φ****′_m_* after the combination of the three channels. Because YUV space is used in Figure 5, the color of the map in Figure 5d is pseudo-color and different from the RGB image. The red part in Figure 5d is the high brightness region, which corresponds to the white highlight part of the general RGB image, and the green part in Figure 5d corresponds to the low brightness region of the RGB image. The channels of Y, U, and V display different information, respectively. After combining the three channels, both color information and fused image information are displayed.

**Figure 6:**
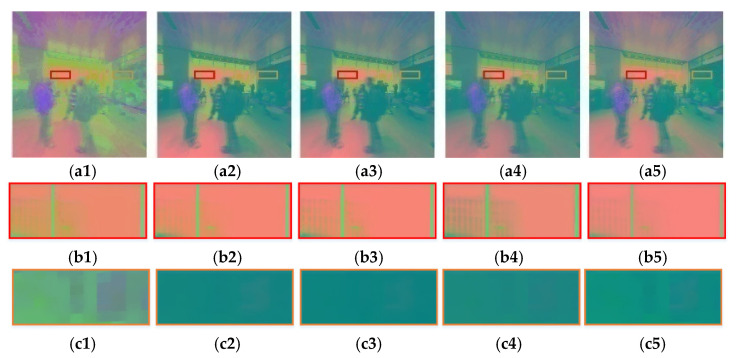
The binocular fusion maps of the viewport images processed by five TMOs (from left to right is DurandTMO [46], Khan [52], KimKautz [53], Reinhard02 [54] and Reinhard05 [55]). (**a1**–**a5**) Binocular fusion maps; (**b1**–**b5**) Partially enlarged maps of highlighted regions, corresponding to the red rectangular boxes in (**a1**–**a5**); (**c1**–**c5**) Partially enlarged maps of low–dark regions, corresponding to the orange rectangular boxes in (**a1**–**a5**).

Figure 6a1–a5 show the binocular fusion maps of the viewport processed by five TM operators (from left to right is DurandTMO [46], Khan [52], KimKautz [53], Reinhard02 [54] and Reinhard05 [55]), where the high brightness and low brightness regions are marked with red rectangular boxes and orange rectangular boxes, respectively. Figure 6b1–b5 and Figure 6c1–c5, respectively, correspond to their local enlarged maps. It can be seen that different TM operators have different degrees of information retention in the global color and local high/low brightness regions, especially in the window regions of Figure 6b1–b5. Accordingly, brightness-based segmentation can be performed, and differentiated feature extraction can be performed according to the characteristics exhibited by different brightness regions.

To sum up, the brightness segmentation based on the maximum entropy threshold [56] is performed on the fusion map ***Φ****′_m_*, and the high brightness region ***Φ****′_m,H_*, the low brightness region ***Φ****′_m,L_* and the middle brightness region ***Φ****′_m,M_* can be also obtained. The maximum entropy threshold segmentation can relatively completely separate the three different brightness regions, and is in line with the subjective brightness perception of the human eyes.

Some studies have shown that visual perception of bright/dark information is unbalanced [57], so different feature extraction schemes should be designed for high/low brightness regions of TM-distorted images; for example, combining the functions of cones and rod cells on retinal photoreceptors, as cone cells mainly work in bright regions and can recognize texture information, while in dark regions, rod cells will work and recognize contour features. Thus, texture features can be extracted for high brightness regions, and contour features can be extracted for low brightness regions. To ensure information integrity, chrominance features are extracted in the middle brightness region. In particular, the process from brightness segmentation to feature extraction of texture, contour and chromaticity is in line with the human vision system, that is, the V1 area perceives primary luminance features, the V2 area perceives high-level features such as texture and shape, and the V4 area perceives color information delivery mechanism.

For the high brightness region ***Φ****′_m,H_* of the binocular fusion map ***Φ****′_m_*, its gray-gradient co-occurrence matrix (GGCM) is calculated to characterize its texture features. GGCM combines image’s gray-scale elements and gradient elements. It can clearly describe the statistical characteristics of gray-scale values and gradients of each pixel in an image and the spatial position relationship between each pixel and its neighboring pixels. Here, gradient is added to the gray level co-occurrence matrix to make it accurate for describing texture. Let ***Y*** denote the gray-scale map of ***Φ****′_m,H_*, and ***G_Y_*** denote the gradient map of ***Y***. First, gradient normalization ***G****′**_Y_*** is performed on ***G_Y_*** as follows:

(17) $${G^{\prime }}_{Y}(i,j)=\mathrm{INT}[(G_{Y}(i,j)-G_{Y\min})/(G_{Y\max}-G_{Y\min})(L_{g}-1)]$$

where INT(·) is the rounding function, (*i*,*j*) is the position of pixel in ***G_Y_***, *G_Ymax_* and *G_Ymin_* are the maximum and minimum values of ***G_Y_***, respectively, and *L_g_* is set to 32.

Similarly, gray-scale normalization ***Y****′* is performed on ***Y*** as follows:

(18) $$Y^{\prime }(i,j)=\mathrm{INT}[(Y(i,j)-Y_{\min})/(Y_{\max}-Y_{\min})(L_{x}-1)]$$

where *Y_max_* and *Y_min_* are the maximum and minimum values of ***Y***, and *L_x_* is set to 32.

Let ***P*** denote a GGCM, then, its normalized GGCM, ***P_N_***, can be expressed as follows:

(19) $$P_{N}(a,b)=P(a,b)/\sum_{a}\sum_{b}P(a,b)$$

where *a* and *b* are the pixel values at the same position in ***Y****′* and ***G****′**_Y_***, respectively.

Based on GGCM, a series of statistical features are derived to describe the image’s texture features. Here, five important statistical measures are adopted to describe texture features, namely, gray mean square deviation *T*_1_, gradient mean square deviation *T*_2_, gray entropy *T*_3_, gradient entropy *T*_4_ and mixed entropy *T*_5_, and calculated as follows:

(20) $$T_{1}=\sqrt{\sum_{a=1}^{L_{x}}{\left(a-\mu_{Y^{\prime }}\right)}^{2}(\sum_{b=1}^{L_{g}}P_{N}(a,b))}$$

(21) $$T_{2}=\sqrt{\sum_{b=1}^{L_{g}}{\left(b-\mu_{G^{\prime }}\right)}^{2}(\sum_{a=1}^{L_{x}}P_{N}(a,b))}$$

(22) $$T_{3}=-\sum_{a=1}^{L_{x}}\left(\sum_{b=1}^{L_{g}}P_{N}(a,b)\right)\cdot \log(\sum_{b=1}^{L_{g}}P_{N}(a,b))$$

(23) $$T_{4}=-\sum_{b=1}^{L_{g}}\left(\sum_{a=1}^{L_{x}}P_{N}(a,b)\right)\cdot \log(\sum_{a=1}^{L_{x}}P_{N}(a,b))$$

(24) $$T_{5}=-\sum_{a=1}^{L_{x}}\left(\sum_{b=1}^{L_{g}}P_{N}(a,b)\right)\cdot \log(P_{N}(a,b))$$

where *μ****_Y_****_′_* and *μ****_G_****_′_* are the mean values of ***Y****′* and ***G****′**_Y_***, respectively.

Then, *T*_1_, *T*_2_, *T*_3_, *T*_4_ and *T*_5_ are taken as the texture features ***f****_GGCM_* of the high brightness region ***Φ****′_m,H_*.

For the low brightness region ***Φ****′_m,L_* of ***Φ****′_m_*, its narrow-sense contour feature is extracted. The contour feature is usually an efficient representation of the shape of an object in an undistorted image. However, considering that the encoding distortion in the low brightness region appears as the block effect, which visually represents the rectangular outline information. The narrow-sense contour feature is defined here to describe the visual distortion phenomenon of the object shape and the block effect. Because different TMOs change the image information in their own ways, when performing brightness segmentation on the TM image, it first appears that the segmented edges are inconsistent; second, different TMOs will cause different visual effects of encoding distortion in low brightness region. In Figure 6, the encoding distortion of DurandTMO in Figure 6c1 is more obvious in the low brightness region, and its block effect outline is more obvious, resulting in drastic changes in the gray-scale value of edge pixels; the block effect caused by Reinhard05 in Figure 6c2 is the second. The results of the other three TMOs in Figure 6c1–c5 are visually similar. Based on this, the energy of gradient (EoG) function is used to measure this change. Let *E_og_* denote EoG of ***Φ****′_m,L_*, then, it is calculated as follows:

(25) $$E_{og}=\sum_{x}\sum_{y}\left\{{({\Phi^{\prime }}_{m,L}(x+1,y)-{\Phi^{\prime }}_{m,L}(x,y))}^{2}+{({\Phi^{\prime }}_{m,L}(x,y+1)-{\Phi^{\prime }}_{m,L}(x,y))}^{2}\right\}$$

Here, the average value ${\bar{Ε}}_{og}$ of *E_og_* is defined as the narrow-sense contour feature. Considering that the block effect at different resolutions may be different, ***Φ****′_m,L_* is down-sampled with three scales, and the ${\bar{Ε}}_{og}$ values at the four scales are taken as the final narrow-sense contour features ***f****_EOG_* of the low brightness region ***Φ****′_m,L_*. Obviously, ***f****_EOG_* is a multiscale feature vector.

Table 3 lists the ${\bar{Ε}}_{og}$ values of the five TMOs at four scales of ***Φ****′_m,L_*. Obviously, the ${\bar{Ε}}_{og}$ values with DurandTMO [46] and Reinhard05 [55] are in the top two positions, followed by Reinhard02 [54]; while Khan [52], KimKautz [53] are numerically similar. According to Figure 6c1–c5, DurandTMO and Reinhard05 lead to an obvious block effect visually; for Reinhard02, a small amount of block effect can be observed, while the block effect can be hardly observed for Khan [52], KimKautz [53]. It means that it is effective to use EoG to measure narrow-sense contour features, which is consistent with subjective perception.

For the middle brightness region ***Φ****′_m,M_*, chrominance statistical features are extracted. Considering that the image distortion changes the natural scene statistical distribution of its mean subtracted contrast normalized (MSCN) coefficients, the asymmetric generalized Gaussian distribution (AGGD) model can fit this distribution, and the difference in the fitting parameters represents the statistical distribution changes. Thus, the four parameters after AGGD fitting, that is, mean *δ_m_*, shape parameter *θ_m_*, left scale parameter $\phi_{l}^{2}$ and right scale parameter $\phi_{r}^{2}$, are used as chrominance statistical features. The chrominance statistical features of the U and V channels are used as the natural statistical features ***f****_AGGD_* of the middle brightness region ***Φ****′_m,M_*.

In summary, the above features extracted are expressed as the texture features ***f****_GGCM_*, narrow-sense contour features ***f****_E__o__G_* and natural scene statistical features ***f****_AGGD_*. These features are all based on viewport images, so according to Equation (12), they are aggregated according to the viewport saliency to obtain ***F****_GGCM_*, ***F****_E__o__G_* and ***F****_AGGD_*, respectively. Thus, the final features extracted by the binocular fusion model are ***F****_fus_* = {***F****_GGCM_*, ***F****_EOG_*, ***F****_AGGD_*}.

(3) Binocular difference map and feature extraction

Because the left and right views of HSOI’s viewpoints are processed by the same TMO for viewing HSOI by user’s HMD with SDR, generally speaking, there is no new color difference between the left and right views after TM. Whereby, the binocular difference information is directly described in the gray-scale channel. Based on joint image filtering, let ${\hat{H}}_{L,m}$ and ${\hat{H}}_{R,m}$ be the viewport’s left and right views after the joint image filtering of their gray-scale channel, respectively. ${\hat{H}}_{L,m}$ and ${\hat{H}}_{R,m}$ can be regarded as image contents that can be initially fused during binocular matching. The absolute difference maps produced by subtracting the distorted viewport images (***V****_L_*_,*m*_, ***V****_R_*_,*m*_) from their jointly filtered viewport images are taken as the left and right monocular difference maps (***MD****_L_*_,*m*_, ***MD****_R_*_,*m*_), where $MD_{L,m}=|V_{L,m}-{\hat{H}}_{L,m}|$ and $MD_{R,m}=|V_{R,m}-{\hat{H}}_{R,m}|$. The monocular difference map represents the information that cannot be fused between left and right views, and the information that cannot be fused may lead to binocular competition.

The related studies [58] showed that binocular competition occurs in all contrast, and the higher the contrast of a monocular stimuli, the stronger its dominant perception. Therefore, a contrast map is calculated as the competition factor, which weights the left and right monocular difference maps (***MD****_L_*_,*m*_, ***MD****_R_*_,*m*_) to obtain the binocular difference map ***BD****_m_*. As mentioned, the contrast map is expressed as ***C*** = ***σ_e_***/(***μ_e_*** + *ε*). Let ***CE****_L_*_,*m*_ and ***CE****_R_*_,*m*_ be the contrast maps of ***MD****_L_*_,*m*_ and ***MD****_R_*_,*m*_, respectively, then, the binocular difference map ***BD****_m_* is computed as follows:

(26) $$BD_{m}=\frac{CE_{L,m}}{CE_{L,m}+CE_{R,m}+\varepsilon }\cdot MD_{L,m}+\frac{CE_{R,m}}{CE_{L,m}+CE_{R,m}+\varepsilon }\cdot MD_{R,m}$$

Considering that the binocular difference map mainly represents the contour information dominated by structure, discrete multidimensional differentiators [59] are used to characterize the binocular difference map ***BD****_m_*, in which five types of derivative maps on ***BD****_m_* are computed, that is, first-order horizontal derivative map ***g****_x_*, first-order vertical derivative map ***g****_y_*, second-order horizontal derivative map ***g****_xx_*, second-order vertical derivative map ***g****_yy_* and second-order mined derivative map ***g****_xy_*.

**Figure 7:**
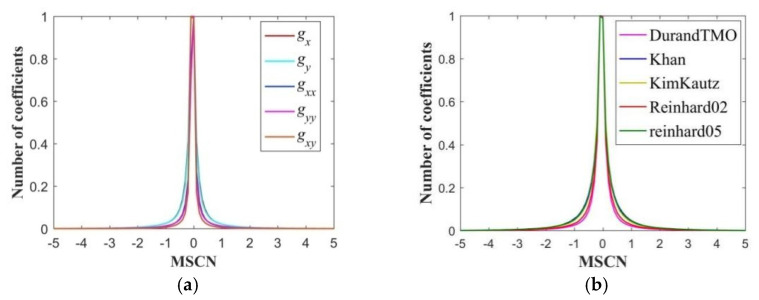
MSCN coefficient distribution curves of derivative maps. (**a**) MSCN coefficient distribution curves of the five derivative maps of the HSOI, which is first compressed by JPEG XT and then processed by DurandTMO, (**b**) MSCN distribution curves of ***g****_x_* of the HSOI which is first compressed by JPEG XT and then processed by the five TMOs.

Figure 7a shows the MSCN coefficient distribution curves of the five derivative maps of the HSOI which is first compressed by JPEG XT and then processed by DurandTMO. Figure 7b shows the MSCN distribution curves of ***g****_x_* of the HSOI which is first compressed by JPEG XT and then processed by the five TMOs. In order to describe their MSCN coefficient distribution, the generalized Gaussian distribution (GGD) model, $f(x;\alpha_{g},\sigma_{g}^{2})$, is used for fitting, where $\alpha_{g}$ and $\sigma_{g}^{2}$ represent the shape and variance parameters of the GGD model, respectively.

For ***BD****_m_*, the shape and variance parameters of the GGD model of its five types of derivative maps are extracted as the binocular difference features, and expressed as ***f****_dif_*. With Equation (12), ***f****_dif_* is further weighted by viewport significance, and the aggregated features are generated as ***F****_dif_*.

### 2.5. Feature Screening and Quality Prediction

As mentioned above, a total of 133 dimensional features are extracted and denoted as ***F****_HSOI_* = {***F****_ec_*, ***F****_corr_*, ***F****_clbp_*, ***F****_st_*, ***F****_fus_*, ***F****_dif_*}, as shown in Table 4. The proposed metric designs a variety of feature extraction processes for visual perception of the distorted HSOI, however, there may be redundancy in ***F****_HSOI_*. Therefore, by performing feature screening on ***F****_HSOI_*, we can obtain a screened feature vector that is conducive to achieve the best performance, and seek a balance between the feature dimension and objective quality evaluation performance.

The Gini coefficient of random forest [60] can be used to calculate the contribution of a single feature on each decision tree. The average contribution of all decision trees is the contribution value of this feature and also the weight of this feature in regression prediction. The contribution values of all feature vectors are arranged in descending order, and the features with high contribution values are selectively retained for feature screening. The screened feature vector after feature screening is recorded as ***F****_FS_HSOI_*.

The screened feature vector ***F****_FS_HSOI_* is used as input, and the random forest model *R_F_*(·) is used as the quality regression model to realize the mapping from the screened feature vector to predict the objective quality score of distorted HSOI, and expressed as follows:

*Q_HSOI_* = *R_F_*(***F****_FS_HSOI_*) (27)

## 3. Experimental Results and Analyses

**Figure 8:**
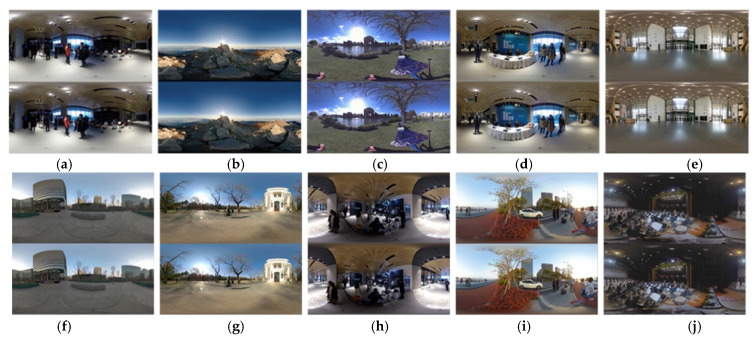
(**a**–**j**) Ten scenes in the HSOID database.

To verify the effectiveness of the proposed HSOIQA metric, it is tested on the HDR stereoscopic omnidirectional image database (HSOID) [45]. The HSOID includes ten scenes (i.e., indoor, outdoor and night scenes), Figure 8 shows ten scenes with the ERP format in the HSOID, which are generated from the stereoscopic omnidirectional video dataset VRQ-TJU [61], the SOLID dataset [62], NBU-SOID dataset [28] and the YouTube. The HSOID includes nine JPEG XT encoding distortion levels and five kinds of TM distortions resulted from five different TMOs; thus, a total of 450 distorted images are contained.

For the distorted HSOI with the JPEG XT encoding, nine encoding distortion levels are designed, including five asymmetric encoding distortions and four symmetric encoding distortions. While for TM distortion, five representative TMOs are selected to map HSOI to SDR domain, and five TMOs are DurandTMO [46], Khan [52], KimKautz [53], Reinhard02 [54] and Reinhard05 [55], respectively. The distortion-produced process includes JPEG XT coding and TM. JPEG XT technology decomposes HSOI into the base layer and extension layer. Distortion degree of the base layer is determined by one quality factor *q*, and that of the extension layer is determined by another quality factor *Q*. Four quality factor pairs are set for compressing each view of HSOI with the JPEG XT, and the corresponding distortion level is represented by L1, L2, L3 and L4, respectively, from high to low. The four quality factor pairs (*q*, *Q*) are set to (16, 9), (30, 19), (50, 30) and (90, 72), which correspond to L1, L2, L3 and L4, respectively. Considering the stereoscopic perception of HSOI, the left view or right view is, respectively, compressed with one of the four quality factor pairs to produce nine distortion levels, including four symmetric encoding distortion levels and five asymmetric encoding distortion levels, as shown in Table 5. Thirty subjects were invited to participate in the subjective experiment. The subjective experiment was conducted in Ningbo University. A total of 30 subjects aged between 20 and 30 years old, including male and female, professional and non-professional, were invited to participate in the experiment to ensure that the experimental data was completely authentic and reliable. The experimental equipment is the HTC Vive Pro HMD with a monocular (left view or right view) viewport resolution of 1440 × 1600 and its FoV angle is 110°. In the experiments, a rotatable seat was provided for the subject, and the subjects wore the HMD to view the omnidirectional images from various viewing angles. Oral guidance was given first before the subjective evaluation, and the subjects were informed of the characteristics of JPEG distortion, TM distortion, mixed distortion and the relevant information such as the scoring standard. To prevent the subjects from being unable to give accurate scores due to discomfort such as visual fatigue and dizziness, when the subjects finished evaluating 45 test images, they rested for 10 min to improve the reliability of the scores as much as possible [45].

**Figure 9:**
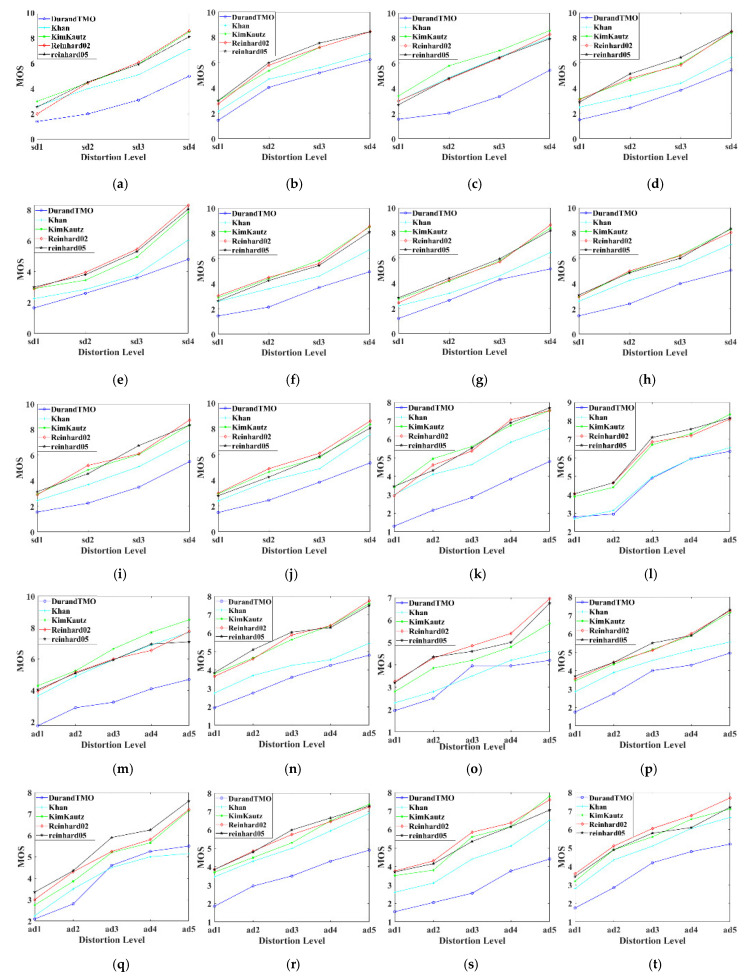
MOS values of the distorted HSOIs in the HSOID dataset. (**a**–**j**) are symmetric coding distortion, corresponding to (**a**–**j**); (**k**–**t**) are asymmetric coding distortion, corresponding to (**a**–**j**).

The subjective score has nine quality levels, and the higher the score, the better the quality. The outliers in the subjective scores were eliminated in strict accordance with the screening criteria, and the average value of the remaining effective scores of each HSOI was taken as its MOS value [45]. Figure 9a–j show MOS values of symmetric distorted HSOIs of ten scenes, and Figure 9k–t are those of asymmetric distorted HSOIs. Here, the MOS curves shown in Figure 9 are the same as the results in [45], where the subjective scoring values of the distorted HSOIs in the HSOID are displayed in different visual ways. It can be found that: (1) For symmetric/asymmetric distortion in all scenarios, the MOS values of the HSOIs processed with DurandTMO and Khan operators are relatively low, while the other three TMOs have little difference. This may be because DurandTMO will “create an illusion” in color, and the TM distortion with Khan is more easily perceived. (2) For symmetric distortion, the MOS values of all scenes increase with the reduction of distortion, and the change trend of MOS among different levels is relatively “steep”, which indicates the rationality of quality factor setting. (3) For asymmetric distortion, the change of MOS values between ad1–ad2 is gentler than those among ad2–ad4–ad5, as shown in Figure 9l,q. Referring to Table 5, it can be found that when the distortion level of the left view is L1 and the distortion level of the right view changes from L3 to L4, the human eyes are not sensitive to this change. When the distortion level of the right view is L4 and that of the left view changes from L1 to L3, the MOS values increase rapidly. This phenomenon is consistent with the fact that JPEG XT coding belongs to information additive distortion, and generally the party with more serious distortion is dominant, and this phenomenon will exist even after TM processing.

In the experiments, the random forest model is used to complete the regression prediction task, and the *K*-fold cross validation is used to divide the test and training sets. Specifically, the HSOID database is divided into *K* subsets, where *K* = 10, corresponding to the number of scenes in the dataset. All images with the same scene form a subset to ensure that the training set does not overlap with the test set. The model trained using the *K* − 1 subset is tested on the remaining subset, iterating from the first scenario until all scenarios are traversed. Finally, the average performance of the *K* cross validation is reported. The accuracy of the proposed metric is evaluated based on three classic indexes: PLCC, SROCC and RMSE. PLCC is the correlation between subjective and objective scores, and SROCC is the monotonicity correlation index between two ordered variables. Both values are between [−1,1], and the closer the absolute value is to one, the higher the accuracy of the regression task is, and the closer the RMSE is to zero, the better.

Here, the relevant results of the influence of feature screening on the performance of the proposed metric are first discussed. Then, the proposed metric is compared with several representative 2D-IQA metrics as well as some blind IQA metrics which consider at least one feature of HSOI. The effects of different feature sets involved in the proposed metric are also analyzed, the symmetric/asymmetric distortion is discussed, and the influence of the number of viewports is analyzed.

### 3.1. Feature Screening in the Proposed Metric

**Figure 10:**
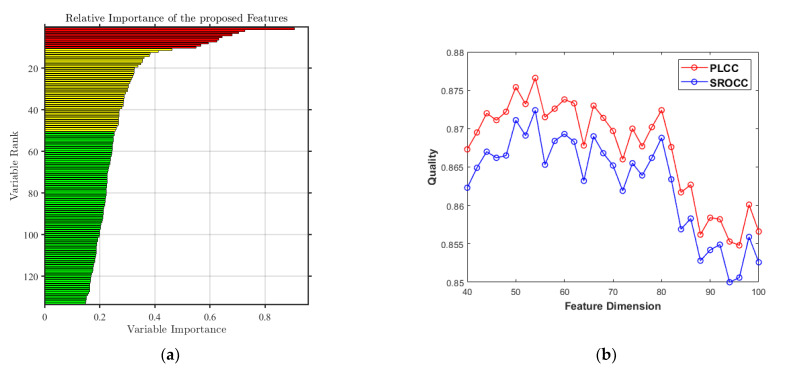
Feature screening. (**a**) Descending arrangement of contribution values of all features. (**b**) Performance of feature vector with different dimensions.

The main purpose of feature screening is to select the feature vector from the features extracted by the proposed perceptual modules, so as to optimize the performance of objective quality metrics. Figure 10a depicts a descending arrangement of contribution values of all extracted features in the proposed metric. As shown in Table 4, the initial extracted feature set has 133 dimensions in total. When the feature dimensions drop to 106 dimensions, the contribution of the remaining features has dropped below 0.2, indicating that most of the extracted features are relatively effective. In order to further select the best feature set, the quality regression is conducted with features of different dimensions to test the objective quality evaluation performance. The results are shown in Figure 10b. Through experiments and verification, when the first 54 features screened according to feature importance are selected, the selected features cover all perceptual modules and achieve the better performance. Thus, the first 54 dimensional features are selected as the final feature set ***F****_FS_HSOI_* in the proposed metric.

### 3.2. Overall Performance of the Proposed Metric

In order to illustrate the effectiveness of the proposed metric, in addition to some representative 2D-IQA metrics, the proposed metric is compared with four types of blind IQA metrics, namely, one SIQA metric (i.e., SINQ [20]), one OIQA metric (i.e., SSP-OIQA [24]), two TM-IQA metrics (i.e., BTMQI [32], BTMIQA [33]), and one SOIQA metric (i.e., Qi’s metric [28]). These four types of metrics involve HSOI’s one or more perception characteristics including binocular perception, OI viewport perception and HDR/TM visual perception. All supervised learning-based metrics are trained by *K*-fold cross validation. To ensure the fairness and reliability of the data, all metrics are tested with the codes released by the corresponding authors.

Table 6 shows the objective assessment results of different metrics on the HSOID dataset, and highlights the best performance indicators in bold. SINQ [20] takes into account the perceptual features of stereovision, SSP-OIQA [24] considers the characteristics of OIs, and two TM-IQA metrics take into account the perceptual features of TM distortion; each of them belongs to one of the perceptual features of HSOI. It is obvious that the TM-IQA metrics (BTMQI and BTMIQA) outperform SINQ; moreover, the PLCC and SROCC of Qi’s metric are almost equal to those of BTMQI; it indicates that TM distortion plays an important role in visual perception of HSOI. This may be because human eyes are extremely sensitive to color changes in TM distortion, and color is an intuitive global attribute. SSP-OIQA uses the SSP format to evaluate the OI quality. The reason for poor performance may be that it does not consider the stereoscopic perception and the TM distortion. It can be found from Table 6 that the performance of the proposed metric is the best, with PLCC and SROCC values reaching 0.8766 and 0.8724, respectively. This is because the proposed metric is based on the characteristics of stereoscopic perception, combined with HDR/TM perception and omnidirectional perception, and utilizes a series of effective feature extraction schemes to evaluate various distortions. Therefore, the proposed metric has better performance of HSOIQA.

### 3.3. Performance Analysis of Different Feature Sets

There are six perceptual feature sets involved in the proposed metric, i.e., ***F****_HSOI_* = {***F****_ec_*, ***F****_corr_*, ***F****_clbp_*, ***F****_st_*, ***F****_fus_*, ***F****_dif_*}, specifically, global color feature ***F****_ec_*, symmetric/asymmetric coding distortion feature ***F****_corr_*, detail contrast feature ***F****_clbp_*, structural feature ***F****_st_*, binocular fusion feature ***F****_fus_* and binocular difference feature ***F****_dif_*. The random forest model is used to train each feature set or combined feature separately, and then its performance is reported, as shown in Table 7, from which the observations can be obtained as follows.

(1) For the proposed six perceptual feature sets, the performance of using global color feature ***F****_ec_* and detail contrast feature ***F****_clbp_* is relatively good. This may be because ***F****_ec_* is combined with the spatial domain and transform domain for feature extraction, and color distortion is one of the obvious distortions in the HSOID dataset. The performance of detail contrast feature ***F****_clbp_* benefits from its combination of contrast and image content in the detail layer. The performance of ***F****_fus_* and ***F****_dif_* based on the BPM is general. This may be because the binocular fusion maps and binocular difference maps of the viewports can be regarded as local images compared with the entire images in ERP format, so that the image content information is relatively small, and the subsequent extracted features can only measure part of the image distortion.
(2) For the MPM, ***F****_clbp_* and ***F****_st_* are the features based on the scene distortion perception. Although the PLCC value of ***F****_st_* itself is only 0.3730, the combination of ***F****_clbp_* and ***F****_st_* has further increased by 0.0219, and the SROCC value by 0.0181 on the basis of ***F****_clbp_*, which is quite impressive. It shows that the design of Laplacian pyramid decomposition combined with image detail contrast and structure is effective. The PLCC value of the global color feature ***F****_ec_* reaches 0.6885, and the addition of ***F****_ec_* increases 0.0654 on the basis of ***F****_clbp_* + ***F****_st_*, while for the SROCC improvement is 0.0640. It implies that measuring color characteristics is very important for HSOIQA. Although the PLCC value of symmetric/asymmetric encoding distortion feature ***F****_corr_* designed for binocular characteristics is only 0.4717, it is further increased by 0.0072 and the SROCC value by 0.0081 on the basis of ***F****_ec_* + ***F****_clbp_* + ***F****_st_*. It can be found that the features extracted by the proposed method for asymmetric distortion in the HSOID dataset are effective and necessary.
(3) For the BPM, the PLCC and SROCC values of the binocular fusion feature ***F****_fus_* are 0.4896 and 0.4673, respectively, while the PLCC and SROCC values of the binocular difference feature ***F****_dif_* are 0.3506 and 0.3033, respectively. The PLCC and SROCC values of ***F****_fus_* + ***F****_dif_* reach 0.5564 and 0.5259, respectively. This means that these two feature sets are complementary, and the binocular perception is also one of the factors that can be considered for HSOIQA.
(4) The PLCC value of the MPM is 0.8525, while that of the BPM is 0.5564; when they are combined, the PLCC value reaches 0.8535. Although the performance of the BPM is general, it further strengthens the prediction ability of the overall model, which means that the MPM and BPM have a positive complementary role. After feature screening, the PLCC and SROCC values of the overall model reach 0.8766 and 0.8724, respectively. It indicates that feature screening removes the redundancy among the initial extracted features and further improves the prediction accuracy of the proposed metric.

### 3.4. Performance Analysison Symmetric/Asymmetric Distortions

For HSOIQA, the binocular perception is one of the important characteristics to be considered. A symmetric/asymmetric distortion measurement model is purposely designed to measure the asymmetric encoding distortion in the HSOID dataset. To verify the effectiveness of the proposed metric, it is compared with the other metrics in Table 6 except the 2D-IQA metrics. The HSOID dataset is divided into the symmetric distorted HSOIs and asymmetric distorted HSOIs to form two sub-datasets so that the relevant metrics can be tested on the two kinds of HSOIs separately. Table 8 lists the performance results of these metrics, where ΔPLCC is the PLCC value of the sub-dataset of the symmetric distorted HSOIs minus the overall PLCC value. ΔPLCC indicates the performance degradation caused by the sub-dataset of the asymmetric distorted HSOIs; and the smaller the ΔPLCC value, the better the performance. In Table 8, the best performance values are shown in bold. It can be found that: (1) The performance of all metrics for the symmetric distorted HSOIs is better than that for asymmetric distorted HSOIs, and the overall performance is between the two ones; and the better the performance of asymmetric distorted HSOIs, the better the overall performance, which implies that asymmetric distortion has to be characterized. (2) For symmetric/asymmetric distortion, all indexes of the proposed metric are significantly better than those of the other metrics, this means that the presented feature extraction schemes for symmetric/asymmetric distortions in the proposed metric is reasonable.

### 3.5. Effect of the Number of Viewports

As described in the Section 2.2, there are totally *M* + 2 viewports sampled from HSOI in the proposed metric, *M* viewports in the equatorial region and two in the bipolar regions. Here, the effect of *M* on the performance of the proposed metric will be tested, where *M* ∈ {4, 6, 8, 10}. When *M ≥* 4, all image information of the equatorial region can be covered. In the experiment, the performance of single feature set as well as the combined feature sets under different viewport numbers are compared, that is, ***F****_corr_*, ***F****_clbp_*, ***F****_st_*, ***F****_fus_*, ***F****_dif_*, ***F****_V_* (***F****_V_* = ***F****_corr_* + ***F****_clbp_* + ***F****_st_* + ***F****_fus_* + ***F****_dif_*), all initial extracted feature sets ***F****_HSOI_* and the screened feature vector ***F****_FS_HSOI_*. Table 9 shows the experimental results with respect to different *M*. The best performance of the same feature set is shown in bold, and the optimal number is the number of times that the corresponding *M* achieves the best performance highlighted with bold. It can be found from Table 9 that when *M* is 8, the optimal number is 17, in which the viewport model and overall performance are the best. Therefore, the total number of viewports is finally determined to be 10, including 8 viewports in the equatorial region and 2 viewports sampled in the polar region. On the other hand, the difference in viewport performance may be due to the redundant information brought by overlapping viewport sampling and the sensitivity of the extracted features to viewport content.

## 4. Conclusions

From the perspective of perception of high dynamic range (HDR) stereoscopic omnidirectional vision system, a visual perception based blind HDR stereoscopic omnidirectional image (HSOI) quality assessment metric has been proposed in this paper. The proposed metric can be divided into two main modules, that is, monocular perception module, and binocular perception module. For the monocular perception module, firstly, the projection format of HSOI is transformed, and then the metric of combining spatial domain and discrete cosine transform domain based on antagonistic channel is designed to measure the global color distortion. Secondly, to measure the symmetric/asymmetric distortion, the absolute correlation coefficient and correlation distance of the left and right views are calculated based on multiscale retinex decomposition; then, considering the characteristics of the indoor, outdoor and night scenes, the detail contrast features and structural features are extracted with Laplacian pyramid decomposition. In the binocular perception module, based on joint image filtering, the binocular fusion map and binocular difference map are calculated. Further, brightness segmentation is performed based on the binocular fusion map, so that the texture, narrow-sense and chromaticity statistical features can be extracted separately for the high, low and middle brightness regions. This process conforms to the information transmission mechanism of the V1, V2 and V4 areas of the human vision system. For binocular difference map, the derivative map is calculated and natural statistical features are extracted. The effectiveness of the proposed metric is compared and analyzed on the HSOID dataset. The experimental results show that the proposed metric is an effective HSOI quality evaluator. In this work, we have presented a comprehensive analysis and empirical study on the problem of HSOI quality assessment, and proposed a new metric. The experimental results also verify its effectiveness. In future work, the performance of HSOI quality evaluation can be improved in two aspects. Firstly, we will further explore the mechanism whereby the visual system perceives the HSOI. Meanwhile, compared with hand-crafted feature extraction, the learning-based feature extraction can be more consistent with the process of the brain processing information. Therefore, a deep learning-based method can be integrated into the evaluation model for performance improvement. In future work, we will consider combining deep learning and visual perception to propose a new network to improve the performance of HSOIQA.

## Figures and Tables

**Table 1:** *C_A_* and *C_D_* of distorted viewport’ left and right views processed by DurandTMO under encoding with 9 distortion levels.

Distortion Level	Absolute Correlation Coefficient *C_A_*				Correlation Distance *C_D_*				Correlation Degree
	0°	45°	90°	135°	0°	45°	90°	135°	
(L1, L3)	0.8709	0.8913	0.8876	0.8985	0.1291	0.1087	0.1124	0.1015	very strong correlation
(L1, L4)	0.4713	0.5386	0.4720	0.5588	0.5287	0.4614	0.5280	0.4412	moderately strong correlation
(L2, L3)	0.9638	0.9735	0.9709	0.9755	0.0362	0.0265	0.0291	0.0245	very strong correlation
(L2, L4)	0.6695	0.7218	0.6650	0.7330	0.3305	0.2782	0.3350	0.2670	strong correlation
(L3, L4)	0.8427	0.8631	0.8310	0.8689	0.1573	0.1369	0.1690	0.1311	very strong correlation
(L1, L1)	0.9991	0.9992	0.9992	0.9994	0.0009	0.0008	0.0008	0.0006	very strong correlation
(L2, L2)	0.9997	0.9994	0.9993	0.9995	0.0003	0.0006	0.0007	0.0005	very strong correlation
(L3, L3)	1.0000	1.0000	0.9999	0.9999	0.0000	0.0000	0.0001	0.0001	very strong correlation
(L4, L4)	0.9998	0.9998	0.9997	0.9997	0.0002	0.0002	0.0003	0.0003	very strong correlation

**Table 2:** Relationship between *C_A_* and correlation degree.

Absolute Correlation Coefficient *C_A_*	Correlation Degree
0.8–1.0	very strong correlation
0.6–0.8	strong correlation
0.4–0.6	moderately strong correlation
0.2–0.4	weak correlation
0.0–0.2	very weak or no correlation

**Table 3:** Average EoG values of five TMOs at four scales.

Scales	DurandTMO [46]	Khan [52]	KimKautz [53]	Reinhard02 [54]	Reinhard05 [55]
Full resolutions	164.9714	23.5691	26.1970	31.9404	50.2103
downsampling 1	172.6434	24.0845	25.9556	32.3332	49.0806
downsampling 2	274.2219	36.4036	37.8075	49.0397	71.1724
downsampling 3	429.1500	53.0810	53.3329	72.3733	101.0040

**Table 4:** The features extracted in the proposed metric.

Feature Extraction Model	Feature Representation	Dimensions
Monocular Perception Module (MPM)	Global color features ***F****_ec_*	14
	Symmetric/asymmetric distortion features ***F****_corr_*	24
	detailed features ***F****_clbp_*	60
	structural features ***F****_st_*	8
Binocular Perception Module (BPM)	binocular fusion features ***F****_fus_*	17
	binocular difference features ***F****_dif_*	10
All	$F_{HSOI}$	133

**Table 5:** Quality factors of nine encoding distortion levels.

Distortion Type	Symmetric Encoding Distortion			Asymmetric Encoding Distortion		
	Symbol	Left View	Right View	Symbol	Left View	Right View
Distortion level	sd1	L1	L1	ad1	L1	L3
	sd2	L2	L2	ad2	L1	L4
	sd3	L3	L3	ad3	L2	L3
	sd4	L4	L4	ad4	L2	L4
	-	-	-	ad5	L3	L4

**Table 6:** Objective assessment results of different metrics on the HSOID dataset.

Type of Metrics	Metrics	PLCC	SROCC	RMSE
2D-IQA	OG-IQA [9]	0.7741	0.7649	1.1595
	GWH-GLBP [10]	0.6677	0.6652	1.3634
	BRISQUE [11]	0.7273	0.7230	1.2571
	IL-NIQE [12]	0.5452	0.5424	1.5354
	dipIQ [13]	0.6290	0.6193	1.4238
	BMPRI [14]	0.4837	0.4495	1.6030
	SISBLIM [15]	0.5928	0.5725	1.4750
SIQA	SINQ [20]	0.6804	0.6732	1.3422
OIQA	SSP-OIQA [24]	0.6413	0.6339	1.4054
TM-IQA	BTMQI [32]	0.7720	0.7690	1.1629
	BTMIQA [33]	0.7067	0.7037	1.2959
SOIQA	Qi′ metric [28]	0.7614	0.7532	1.1873
HSOIQA	Proposed	**0.8766**	**0.8724**	**0.8814**

**Table 7:** Performance of different feature sets and their combination.

Feature Extraction Mode	Features	PLCC	SROCC	RMSE
Single feature set	***F*** *_ec_*	0.6885	0.6685	1.3283
	***F*** *_corr_*	0.4717	0.3494	1.6150
	***F*** *_clbp_*	0.7580	0.7572	1.1946
	***F*** *_st_*	0.3730	0.3591	1.6994
	***F*** *_fus_*	0.4896	0.4673	1.8525
	***F*** *_dif_*	0.3506	0.3033	1.7153
Monocular perception	***F****_clbp_* + ***F****_st_*	0.7799	0.7753	1.1464
	***F****_ec_* + ***F****_clbp_* + ***F****_st_*	0.8453	0.8393	0.9786
	***F****_corr_* + ***F****_ec_* + ***F****_clbp_* + ***F****_st_*	0.8525	0.8474	0.9753
Binocular perception	***F****_fus_* + ***F****_dif_*	0.5564	0.5259	1.5219
All	$F_{HSOI}$	0.8535	0.8488	0.9545
All with feature screening	$F_{FS\_HSOI}$	0.8766	0.8724	0.8814

**Table 8:** Objective quality assessment results of symmetric/asymmetric distortions in the HSOID dataset.

Metrics	Symmetric Distortion			Asymmetric Distortion			Overall			ΔPLCC
	PLCC	SROCC	RMSE	PLCC	SROCC	RMSE	PLCC	SROCC	RMSE	
SINQ	0.7802	0.7760	1.3006	0.5841	0.5671	1.3033	0.6804	0.6732	1.3422	0.0998
SSP-OIQA	0.7502	0.7428	1.3748	0.5349	0.5276	1.3567	0.6413	0.6339	1.4054	0.1089
BTMQI	0.8092	0.8061	1.2217	0.7310	0.7225	1.0957	0.7720	0.7690	1.1629	0.0372
BTMIQA	0.7686	0.7696	1.3300	0.6383	0.6267	1.2361	0.7067	0.7037	1.2959	0.0619
Qi	0.8161	0.8006	1.2015	0.6877	0.6936	1.1657	0.7614	0.7532	1.1873	0.0547
Proposed	**0.9086**	**0.8972**	**0.8685**	**0.8225**	**0.8172**	**0.9131**	**0.8766**	**0.8724**	**0.8814**	**0.0320**

**Table 9:** Objective quality assessment results with respect to different *M* values.

*M*	Feature Type	Features	PLCC	SROCC	RMSE	Optimal Number
4	Viewport-based features	***F*** *_corr_*	0.3789	0.2865	1.6950	0
		***F*** *_clbp_*	0.7542	0.7517	1.2026	
		***F*** *_st_*	0.3607	0.3240	1.7083	
		***F*** *_fus_*	0.3849	0.3162	1.6904	
		***F*** *_dif_*	0.2845	0.2378	1.7559	
		***F*** *_V_*	0.7961	0.7924	1.1085	
	All	***F*** *_HSOI_*	0.8454	0.8404	0.9782	0
	All with FS	***F*** *_FS_HSOI_*	0.8584	0.8523	0.9396	0
6	Viewport-based features	***F*** *_corr_*	0.4423	**0.3646**	1.6427	4
		***F*** *_clbp_*	0.7578	0.7565	1.1951	
		***F*** *_st_*	**0.3978**	**0.3644**	**1.6804**	
		***F*** *_fus_*	0.4605	0.4337	1.6258	
		***F*** *_dif_*	0.2844	0.2443	1.7559	
		***F*** *_V_*	0.7917	0.7889	1.1188	
	All	***F*** *_HSOI_*	0.8478	0.8421	0.9713	0
	All with FS	***F*** *_FS_HSOI_*	0.8688	0.8644	0.9068	0
8	Viewport-based features	***F*** *_corr_*	**0.4717**	0.3494	**1.6150**	11
		***F*** *_clbp_*	**0.7580**	**0.7572**	**1.1946**	
		***F*** *_st_*	0.3730	0.3591	1.6994	
		***F*** *_fus_*	0.4896	**0.4673**	1.8525	
		***F*** *_dif_*	0.3506	**0.3033**	**1.7153**	
		***F*** *_V_*	**0.8051**	**0.8044**	**1.0863**	
	All	***F*** *_HSOI_*	**0.8535**	**0.8488**	**0.9545**	3
	All with FS	***F*** *_FS_HSOI_*	**0.8766**	**0.8724**	**0.8814**	3
10	Viewport-based features	***F*** *_corr_*	0.4292	0.3585	1.6543	3
		***F*** *_clbp_*	0.7532	0.7516	1.2048	
		***F*** *_st_*	0.3740	0.3355	1.6987	
		***F*** *_fus_*	**0.4898**	0.4611	**1.5968**	
		***F*** *_dif_*	**0.3376**	0.2953	1.7240	
		***F*** *_V_*	0.7937	0.7923	1.1141	
	All	***F*** *_HSOI_*	0.8447	0.8401	0.9804	0
	All with FS	***F*** *_FS_HSOI_*	0.8625	0.8568	0.9267	0

## Data Availability

Not applicable.
